# Single-lens dynamic $z$-scanning for simultaneous in situ position detection and laser processing focus control

**DOI:** 10.1038/s41377-023-01303-2

**Published:** 2023-11-17

**Authors:** Xiaohan Du, Camilo Florian, Craig B. Arnold

**Affiliations:** 1 Department of Mechanical and Aerospace Engineering, Princeton University, Princeton, NJ 08544 USA; 2 Department of Systems Engineering, City University of Hong Kong, Hong Kong, China; 3 Institut für Werkstofftechnik, Universität Kassel, 34125 Kassel, Germany; 4 Princeton Materials Institute, Princeton University, Princeton, NJ 08544 USA

**Keywords:** Imaging and sensing, Adaptive optics, Laser material processing, Optical sensors

## Abstract

Existing auto-focusing methods in laser processing typically include two independent modules, one for surface detection and another for $z$-axis adjustment. The latter is mostly implemented by mechanical $z$ stage motion, which is up to three orders of magnitude slower than the lateral processing speed. To alleviate this processing bottleneck, we developed a single-lens approach, using only one high-speed $z$-scanning optical element, to accomplish both in situ surface detection and focus control quasi-simultaneously in a dual-beam setup. The probing beam scans the surface along the $z$-axis continuously, and its reflection is detected by a set of confocal optics. Based on the temporal response of the detected signal, we have developed and experimentally demonstrated a dynamic surface detection method at 140–350 kHz, with a controlled detection range, high repeatability, and minimum linearity error of 1.10%. Sequentially, by synchronizing at a corresponding oscillation phase of the $z$-scanning lens, the fabrication beam is directed to the probed $z$ position for precise focus alignment. Overall, our approach provides instantaneous surface tracking by collecting position information and executing focal control both at 140–350 kHz, which significantly accelerates the axial alignment process and offers great potential for enhancing the speed of advanced manufacturing processes in three-dimensional space.

## Abstract (web summary)

We develop a high-speed focal scanning method for laser processing that can significantly improve processing times. The innovative single-lens approach uses a high-speed axial scanning lens to detect surfaces and adjust laser focus simultaneously, overcoming the slower speeds associated with surface mapping or other mechanical auto-focusing methods.

## Graphical abstract

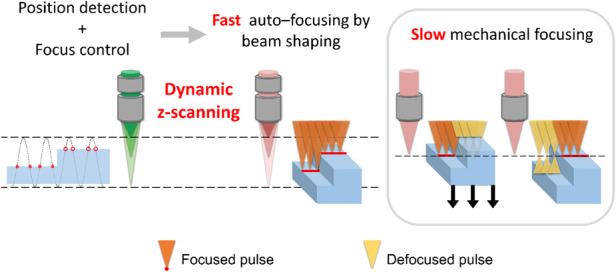

## Introduction

The ability to deliver energy efficiently to designated locations is critical in laser processing, especially for high spatial resolution systems with a tightly focused beam, a small focal voxel, and a narrow depth of field (DOF). Effective focus alignment with synchronous feeding during laser processing can benefit the precision and quality of laser micro-machining^1,2^. The precise alignment of the workpiece’s surface towards the laser focus requires knowing the exact positions of both the focus (${z}_{f}$) and the workpiece (${z}_{s}$). While focus alignment is straightforward for flat surfaces, working with non-flat surfaces is more challenging as it requires the alignment between ${z}_{f}$ and varying ${z}_{s}$ during fast lateral translations. Traditionally, the surface topography of the non-flat surface can be found by ex-situ methods prior to fabrication, such as using interferometry or confocal microscopy. Afterward, a programmed ${xyz}$ stage is used to follow the pre-measured topology. In this way, the non-flat surface is always maintained within the range of the narrow DOF. However, the described method is both time- and labor-intensive, as it involves complex operating procedures and coordination of multiple instruments^3^. Moreover, the ex-situ surface characterization and the axial re-alignment always result in repositioning errors and extended processing times^4^.

The difficulty of axial re-alignment has led to the development of auto-focusing in laser processing of materials. Traditional auto-focusing techniques consist of two independent modules: ‘detection and movement’, i.e., first, in situ and non-destructive detection of ${z}_{s}$; second, $z$ movement by physically moving either the sample or the focusing lens. For the first ‘detection’ module, various research and development works have successfully achieved in situ surface metrology in optical applications such as automatic microscopy^5^, two-photon polymerization^6^, mechanical turning^7^, and laser machining, including direct writing^8,9^, welding^10^, cutting^11^ and bending^12^. While other mechanical, resistance-, and capacitance-based methods exist, the majority of surface detection methods are based on the optical detection of back-reflected light. The focus error or defocusing (${z}_{s}-{z}_{f}$) is determined from the position (triangulation)^5^, intensity^13^, beam size^14,15^, astigmatism^16,17^ of a reflected probing beam, or chromatic aberrations^10,11^, nonlinear harmonics^18^ of process-generated light. Other back-reflected detection methods also adopt differential optical paths^19,20^, interferometry^21,22^, diffractive beam samplers^23^, spatial light modulators^24^, dynamic mechanical scanning^3,25,26^, digital image correlation^12^, or commercial displacement sensors^7,9^. However, most optical metrology with automatic focus alignment still requires the mechanical motion of a $z$ stage attached to the sample^6,14,18,19,22^ or the focusing lens^3,8,10,23–25^ as the second ‘movement’ module in its feedback loop. Even with the advance of the fast mechanical stages and scanning galvo-mirrors, adjusting $z$ position can be up to three orders of magnitude slower than the lateral speed of adjusting $x$ and $y$^27^ due to mechanical acceleration/deceleration. Thus, the process of surface detection and the further re-alignment between ${z}_{f}$ and ${z}_{s}$ is greatly slowed down by mechanical movements.

One way to retrieve the focus quickly after position detection is to incorporate axial varifocal optics^27^, such as deformable mirrors^24,28^, electrical tunable lenses^29^ or liquid crystal devices^30–32^, manipulating the focal position and bypassing delays in acceleration/deceleration from any mechanical motion. However, the response rate of the above varifocal optics is limited to 1 kHz. An ultrafast varifocal lens with a response time on the order of one microsecond, such as a tunable acoustic gradient of refractive index (TAG) lens, is a strong candidate for ultrafast focus sensing and control. The TAG lens^33,34^ is a liquid $z$-scanning lens operating at 0.1–1 MHz, and has been used in multiple imaging^35–40^ and machining^4,41–43^ applications.

Leveraging the ultrafast variable focus of the TAG Lens, we rethink the ‘detection and movement’ strategy and use a single lens as an all-in-one tool to integrate both ‘detection’ and ‘movement’ simultaneously. We first propose a dynamic surface position searching approach based on continuous $z$-scanning of the TAG lens, in which we relate the temporal response of the probing beam’s reflected intensity to surface location. Then we effectively direct the fabrication beam to the designated positions by triggering the fabrication laser at the corresponding phase of the TAG lens based on the probed surface position without any mechanical movement in $z$. Theoretically, the time between surface detection, focus retrieval, and firing of the fabrication laser pulse is within two periods of $z$-scanning at 140–350 kHz, which surpasses any mechanically based re-focusing system.

## Results

### Setup and principles

**Fig. 1:**
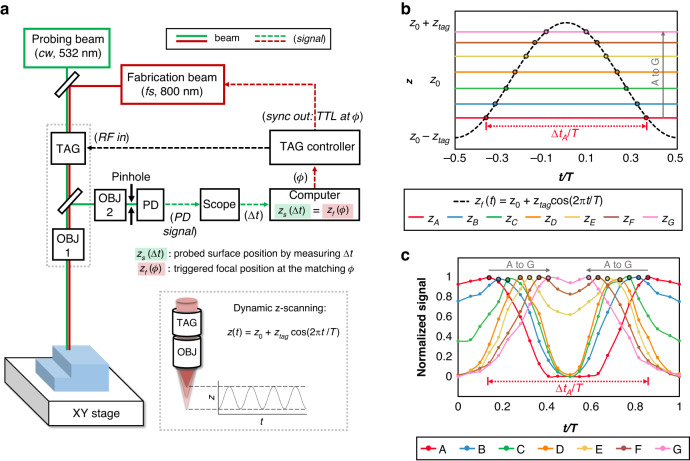
Configuration and principles of the dynamic focusing system. **a** Schematic diagram of the dual-beam setup, with the probing beam path in green and the fabrication beam path in red. The inset plots the oscillating focal position in $z$ due to the oscillating optical power of the TAG lens. The continuous $z$-scanning of the probing beam detects the surface position ${z}_{s}$ by measuring $\varDelta t$ from the photodiode (PD) signal, which corresponds to a matching phase $\phi$ of the fabrication beam such that ${z}_{s}(\varDelta t)={z}_{f}(\phi )$. The fabrication beam is triggered at ${z}_{f}(\phi )$ for precise alignment between the surface and the focus. **b** Plot of one period of focus scanning ${z}_{f}(t)$ and the surface position ${z}_{s}$ at multiple locations (A to G) within the scanning range. **c** Normalized photodiode signal for locations A to G, showing double peaks. The peak-to-peak time interval is acquired as $\varDelta t$

A dual laser beam setup is adopted to implement both position detection and focus control by a $z$-scanning lens. Figure 1a shows the schematic of the designed optical system, consisting of two beam paths, one for probing marked in green, and the other for fabrication marked in red. Unlike a conventional laser processing setup with a fixed focus at ${z}_{0}$, the focal position of the probing beam ${z}_{f}$ continuously oscillates as a function of time $t$ with the inclusion of the TAG lens, as plotted in the inset. The reflected beam passing through the pinhole is detected by a biased photodiode (PD). The proposed surface detection method is based upon the temporal response of the detected reflected light intensity through confocal optics. The varifocal TAG lens shapes the probing beam to a periodic scanning focal spot in $z$, as described by,

1 $${z}_{f}\left(t\right)={z}_{0}+{z}_{{tag}}\cos (2\pi {f}_{{tag}}t)={z}_{0}+{z}_{{tag}}\cos \left(\frac{2\pi t}{T}\right)$$

where ${z}_{0}$ and ${z}_{{tag}}$ are the average and the amplitude of the focus oscillation. ${f}_{{tag}}$ and $T$ are the scanning frequency and the period, which vary around 141 kHz and 7 µs for most experiments in this study. The maximum frequency goes up to 350.8 kHz. For any surface located at ${z}_{s}$ within the oscillation range of ${z}_{f}(t)$, it theoretically aligns with scanning laser focus twice per period at ${t}_{1}$ and ${t}_{2}$ such that,

2 $$\begin{array}{c}{z}_{f}\left({t}_{1}\right)={z}_{f}\left({t}_{2}\right)={z}_{s}\\ {t}_{1,2}=\pm \frac{T}{2\pi }\,{\cos }^{-1}\left(\frac{{z}_{s}-{z}_{0}}{{z}_{{tag}}}\right)\end{array}$$

The alignment between ${z}_{s}$ and ${z}_{f}$ corresponds to a peak in the detected PD signal. Therefore, two peaks in the detected signal would be observed at both ${t}_{1}$ and ${t}_{2}$ per scanning period. Figure 1b plots one period of $z$-scanning and several surfaces located at different locations (A to G) within the scanning range. Figure 1c plots the corresponding normalized PD signals for locations A to G from an experiment numbered as A1. Each surface intersects twice with ${z}_{f}(t)$. For example, the recognized double peaks for location A (red) correspond to the condition of ${z}_{f}\left(t\right)={z}_{A}$. Theoretically, the photodiode signal can also be calculated by a simplified ray transfer matrix model, as detailed in Fig. s1 in the supplementary information. We denote the peak-to-peak time interval $\Delta t$ as the difference between ${t}_{1}$ and ${t}_{2}$.

3 $$\Delta t={t}_{1}-{t}_{2}=\frac{T}{\pi }{\cos }^{-1}\left(\frac{{z}_{s}-{z}_{0}}{{z}_{{tag}}}\right)$$

Therefore, a one-to-one mapping can be established between the time interval $\Delta t$ and the surface position ${z}_{s}$, as

4 $${z}_{s}(\varDelta t)={z}_{0}+{z}_{{tag}}\cos \left(\pi \frac{\Delta t}{T}\right)$$

Equation 4 is the key foundation of our detection method because it describes a linear relation between the surface position ${z}_{s}$ and measured $\cos (\pi \Delta t/T)$ from the PD signal, which can be exploited for building a linear position sensor. The slope ${z}_{{tag}}$ and intercept ${z}_{0}$ are both determined by the parameters of the varifocal TAG lens. Based on the mechanics of the TAG lens, the $z$-scanning amplitude ${z}_{{tag}}$ is linearly proportional to the driving voltage ${V}_{{tag}}$ of the radio frequency (RF) signal from the TAG controller^34,44^, and the $z$-scanning period $T$ is determined by the driving frequency ${f}_{{tag}}$ of the RF signal.

The measured surface position is then converted to a matching phase $\phi$ of the TAG lens such that ${z}_{s}(\Delta t)={z}_{f}(\phi )$ and then communicated to the fabrication component of the optical system, as indicated in red in Fig. 1a. The TAG controller sends a synchronized transistor-transistor logic (TTL) signal at the matching phase $\phi$ to trigger the fabrication laser. Due to the dispersion of the TAG lens media and the difference in optical path, the two constants ${z}_{0}$ and ${z}_{{tag}}$ generally have different values for the two laser beams, as denoted by ${z}_{\mathrm{0,1}}$, ${z}_{{tag},1}$, ${z}_{\mathrm{0,2}}$ and ${z}_{{tag},2}$ for the probing beam and the fabrication beam. The four constants need to be calibrated a priori. Equaling ${z}_{s}$ and ${z}_{f}$ requires the matching phase $\phi$ satisfying the following condition,

5 $$\,\cos (\phi )=\frac{{z}_{{tag},1}}{{z}_{{tag},2}}\cos \left(\pi \frac{\Delta t}{T}\right)+\frac{{z}_{0,1}-{z}_{0,2}}{{z}_{{tag},2}}$$

Equation 5 is a transfer function relating the triggering phase $\phi$ to the measured $\Delta t$ from the photodiode signal. As a result, the synchronized fabrication beam can precisely modify the probed surface without physically moving neither the positioning stage nor the optics. In a simplified case where the two beams have the same beam path and similar wavelength, the two sets of constants are approximately the same, therefore we can find,

6 $$\,\phi =\pi \frac{\Delta t}{T}$$

### In situ position detection

#### Characterization of the in situ position detection method

**Fig. 2:**
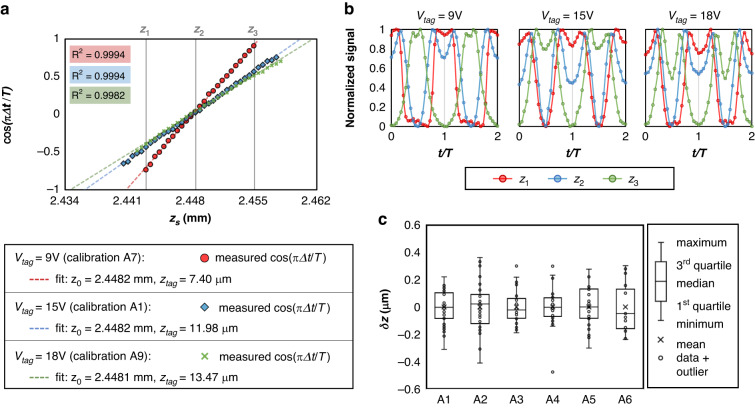
Plot of the measured $\cos (\pi \varDelta t/T)$ versus the real surface position ${z}_{s}$ for OBJ1 $=$ 40$\times$. A linear fit defines the calibrated surface position ${z}_{s}^{{\prime} }$, with the fitted intercept as ${z}_{0}$ and the slope as ${z}_{{tag}}$. Red is for the calibration experiment A7 with ${V}_{{tag}}=\,$ 9 V, blue is for the calibration experiment A1 with ${V}_{{tag}}=$ 15 V, and green is for the calibration experiment A9 with ${V}_{{tag}}=$ 18 V. ${R}^{2}$ of the linear regression is listed. **b** Normalized signal of the photodiode at location ${z}_{1}$, ${z}_{2}$, and ${z}_{3}$ for the three calibration experiments with different driving voltages. **c** Box and whisker chart of the errors ${\delta z=z}_{s}^{{\prime} }-{z}_{s}$ for calibration experiments with OBJ1 $=$ 40$\times$ and ${V}_{{tag}}=$ 15 V (A1-A6)

Multiple calibration experiments of the probing part of the setup are conducted to characterize the linear relationship as described in Eq. 4. We take the inverse of the laser propagation direction as the positive $z$ direction. A silicon wafer is positioned on a mechanical $z$ stage, which moves up towards the beam by a step size of 0.5 µm with OBJ1 $=$ 40$\times$ (NA $=$ 0.65). Details of all calibration experiments are included in Table s1 in the supplementary information. In Fig. 2a, we plot the measured $\cos (\pi \Delta t/T)$ and the recorded $z$ stage position ${z}_{s}$ of three calibration experiments with different scanning amplitudes (${z}_{{tag}}$). The total detection range, 2${z}_{{tag}}$, is linearly proportional to the input voltage of the TAG lens ${V}_{{tag}}$. Red is for the calibration experiment A7 with ${V}_{{tag}}=\,$ 9 V, blue is for the calibration experiment A1 with ${V}_{{tag}}=$ 15 V, and green is for the calibration experiment A9 with ${V}_{{tag}}=$ 18 V. Based on Eq. 4, a linear fit defines the calibrated surface position ${z}_{s}^{{\prime} }$, with the fitted intercept as ${z}_{0}$ and the slope as ${z}_{{tag}}$. The fitted scanning range for driving voltage of 9 V, 15 V, and 18 V is 7.40 µm, 11.98 µm, and 13.47 µm, respectively. Figure 2b displays two periods of the normalized signal for three different $z$ locations (${z}_{1}$, ${z}_{2}$ and ${z}_{3}$) with different driving voltages.

To evaluate the accuracy and repeatability of the developed method under different alignment and driving conditions, we calculate the error between the calibrated position ${z}_{s}^{{\prime} }$ and the real position ${z}_{s}$ by ${\delta z=z}_{s}^{{\prime} }-{z}_{s}$. In Fig. 2c, we plot the distribution of $\delta z$ from the six repeated calibration experiments (A1-A6) with the same driving voltage ${V}_{{tag}}=$ 15 V) in a box and whisker chart. The accuracy of surface sensing is the maximum of errors ($\max \left|\delta z\right|$). We define the linearity error $\epsilon$ as the ratio of accuracy to the total $z$-scanning range, $\epsilon =\frac{\max \left|\delta z\right|}{2{z}_{{tag}}}$. The linearity error ranges from 1.24% to 1.95% in Table s1. Variations in the tabulated ${z}_{0}$, ${z}_{{tag}}$, accuracy, and linearity error are possibly caused by alignment error and fluctuation of TAG lens power under liquid resonance^34^. Based on experiments A1-A6, the average detection range (2${z}_{{tag}}$) with ${V}_{{tag}}=$ 15 V is 23.50 µm, with an average accuracy of 0.35 µm and an average linearity error of 1.49%.

The $z$-scanning range of the focus depends on the optical power of both the TAG lens and the objective lens. It can be approximated as ${z}_{{tag}}\approx {f}_{0}^{2}\left|{op}\right|$ (from Eq. (4) of ref. ^44^), where ${f}_{0}$ is the focal length of the objective lens, and $\left|{op}\right|$ represents the amplitude of the oscillating optical power of the TAG lens. As $\left|{op}\right|$ is linearly related to the driving voltage ${V}_{{tag}}$, the scanning range ${z}_{{tag}}$ is linearly proportional to ${V}_{{tag}}$ and ${f}_{0}^{2}$. By increasing ${f}_{0}$ and utilizing a lower magnification lens (OBJ1), our method can be adapted for a larger detection range. Table s2 in the supplementary information lists the conducted ten calibration experiments with OBJ1 $=$ 4$\times$ (NA $=$ 0.15), where we perform similar analyses as in Table s1. As OBJ1 is changed from 40$\times$ to 4$\times$, the average detection range (2${z}_{{tag}}$) with ${V}_{{tag}}=$ 15 V is extended from 23.5 µm to 1.39 mm, with an average accuracy of 0.026 mm and an average linearity error of 1.87%. In addition, in calibration experiment B10 in Table s2, we operate the TAG lens at a relatively higher frequency of 350.8 kHz. By changing the driving conditions (frequency or amplitude) of the TAG lens and the optical power of the pairing objective lens, we demonstrate that the TAG lens scanning approach is adaptable to measurements of various length scales while keeping the same level of percent error.

Finally, we compare the TAG lens scanning method with other dynamic scanning methods for surface detection, such as mechanical oscillation of a pinhole or a lens, or resonance of a microelectromechanical systems (MEMS) deformable mirror. Table 1 lists the response rate, detection range, accuracy (i.e., maximum of errors), and linearity error of some reported studies. The results for the TAG lens scanning method in this work are highlighted in bold. Representative calibration experiments A6 and B6 are given in Table 1, with additional data given in Tables s1 and s2 for ${V}_{{tag}}=$ 15 V. The dynamic $z$-scanning achieved by the TAG lens is a great substitute for any mechanical oscillation of either a pinhole, a lens, or a deformable mirror, because it substantially accelerates the surface detection process with a much higher response rate while maintaining the percent error, and is adjustable in its detection range.

**Table 1:** Comparison of optical surface detection methods by dynamic $z$-scanning

Working principle	Response rate (Hz)	Detection range	Accuracy ($\max \left|\delta z\right|$)	Linearity error $\epsilon$ (%)
**TAG lens scanning, with 40**${\boldsymbol{\times }}$ **(this work, from A6)**	**140k**	**24.26** **µm**	**0.30** **µm**	**1.24%**
**TAG lens scanning, with 4**${\boldsymbol{\times }}$ **(this work, from B6)**	**140k**	**1.33** **mm**	**0.015** **mm**	**1.10%**
Mechanical oscillation of a pinhole^25^	100	20 µm	0.5 µm	2.50%
Mechanical oscillationof a lens (from Fig. 2b of ref. ^26^)	50	3 mm	0.25 mm	8.33%
Resonance of a MEMS deformable mirror^28^	7k	310 µm	4.4 µm	1.20%

#### Profile measurement of a step surface

**Fig. 3:**
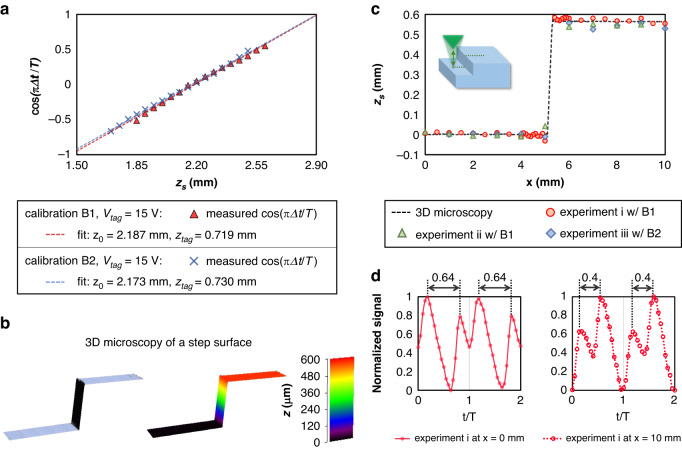
Profile measurement of a step surface. **a** Calibrated surface position ${z}_{s}^{{\prime} }$ with the fitted intercept (${z}_{0}$) and slope (${z}_{{tag}}$) for OBJ1 $=$ 4$\times$ and ${V}_{{tag}}=$ 15 V. Red for the calibration experiment B1, and blue for the calibration experiment B2. **b** 3D microscopy of the step surface obtained from a laser confocal microscope. **c** Measured profile of the step surface with three repeated experiments (i and ii with calibration B1, iii with calibration B2). The dash line plots a reference profile taken by the 3D microscope. **d** Normalized photodiode signal for experiment i at the low surface ($x=$ 0 mm) and the high surface ($x=$ 10 mm). The time interval $\varDelta t$ is measured as 0.64$T$ and 0.4$T$, respectively

Experimentally, we demonstrate a profile measurement of a non-flat, step surface, constructed by adhering two Si wafers of 500 µm thickness with a thin layer of optical glue with unknown thickness. The sample is positioned randomly at $z=$ 2.2 mm for the low surface. OBJ1 is selected to be 4$\times$ to cover the entire step height in the detection range of ${z}_{f}(t)$. The sample is positioned on a stage and moves only in the $x$ direction from 0 to 10 mm. Three sets of experiments are conducted to measure the line profile of the sample under two calibration conditions (B1 and B2, listed in Table s2). Using the fitted parameters from the calibration experiments in Fig. 3a, the measured line profile by the TAG $z$-scanning is compared with an ex-situ reference measurement taken by a laser confocal microscope (Olympus Lext OLS4000), as shown in Fig. 3b. After zeroing the $z$ location of the bottom surface, the measured profiles by TAG scanning and confocal microscope are consistent, as shown in Fig. 3c. Taking the measured ${z}_{s}$ from $x=$ 5.5 mm to $x=$ 10 mm, the average height of step $h$ is 0.572 mm with a standard deviation of 9.57 µm by the TAG lens scanning method (experiment i), versus 0.564 mm with a standard deviation of 9.71 µm by the confocal microscopy. Figure 3d plots the normalized photodiode signal for experiment i at the low surface ($x=$ 0 mm) and the high surface ($x=$ 10 mm). The time interval $\Delta t$ is measured as 0.64$T$ and 0.4$T$, respectively.

### Synchronous focus control

In principle, the liquid resonance of the TAG lens continuously oscillates its lens power, resulting in a continuous scanning focal position ${z}_{f}\left(t\right)$. Alternatively, the focal position can be selected discretely by synchronizing a pulsed laser with a single phase $\phi$ of the TAG lens, as recently reported in^44^.

7 $$\,{z}_{f}(\phi )={z}_{0}+{z}_{{tag}}\cos (\phi )$$

The pulsed laser is triggered by a TTL pulse elevated at the selected single phase $\phi$. The width of the triggering pulse is denoted by ${\delta }_{t}$. ${\delta }_{t}$ is critical in determining both the repetition rate of synchronized pulses and the accuracy of focus control. The repetition rate of the synchronized pulse ${f}_{{sync}}$ can be calculated by ${f}_{{sync}}={f}_{{laser}}\cdot {f}_{{tag}}\cdot {\delta }_{t}$, where ${f}_{{laser}}$ is the laser’s internal repetition rate. In this work, ${\delta }_{t}=$ 1 µs, ${f}_{{laser}}=\,$ kHz, ${f}_{{tag}}=$ 140.1 kHz, and ${f}_{{sync}}=$ 140.1 Hz. The frequency reduction from 1 kHz to 140.1 Hz indicates that only 14% of the internal pulses are triggered in the synchronization mode. Hence, the synchronization method is more powerful when the fabrication laser has a compatible or higher response rate than the TAG lens.

We define the focus control accuracy as the axial width of the focal position when triggered at a specific phase $\phi$. This accuracy can be estimated by multiplying the axial scanning speed with the width ${\delta }_{t}$ of the triggering pulse^44^. The axial scanning speed reaches its minimum at 0° and 180°, while it is maximized at 90°. Consequently, the axial width is also maximized at 90°. To illustrate this, we characterize ${z}_{f}(\phi )$ triggered at various phases $\phi$ from 0°to 180° (Fig. s4a, supplementary information). By performing a least-square fit based on Eq. 7, we obtain ${z}_{{tag}}=-$0.53 mm and ${z}_{0}={f}_{0}-$0.27 mm, with ${f}_{0}$ being the focal length of the objective lens. To account for uncertainty from the triggering pulse width ${\delta }_{t}$, we plot an envelope of ${z}_{f}(\phi )$ in Fig. s4a, adding a $\pm \pi {f}_{{tag}}{\delta }_{t}$ term to its phase. The accuracy of synchronous focus control is then defined as the axial width of this envelope at 90°. For ${z}_{{tag}}=-$0.53 mm, the maximum axial width at $\phi =$ 90° is $\pm$ 0.22 mm, considering ${\delta }_{t}=$ 1 µs.

Based on the measured ${z}_{s}$ by the probing beam, a matching phase $\phi$ which satisfies ${z}_{s}={z}_{f}(\phi )$ can be calculated to trigger the fabrication beam, as described in Eq. 5. In this way, the fabrication beam can effectively modify the surface based on the in situ measurement from the probing beam while neither the positioning stage nor any optical elements are physically moved in $z$ in a single lateral scanning.

#### Comparison of three focusing strategies

**Fig. 4:**
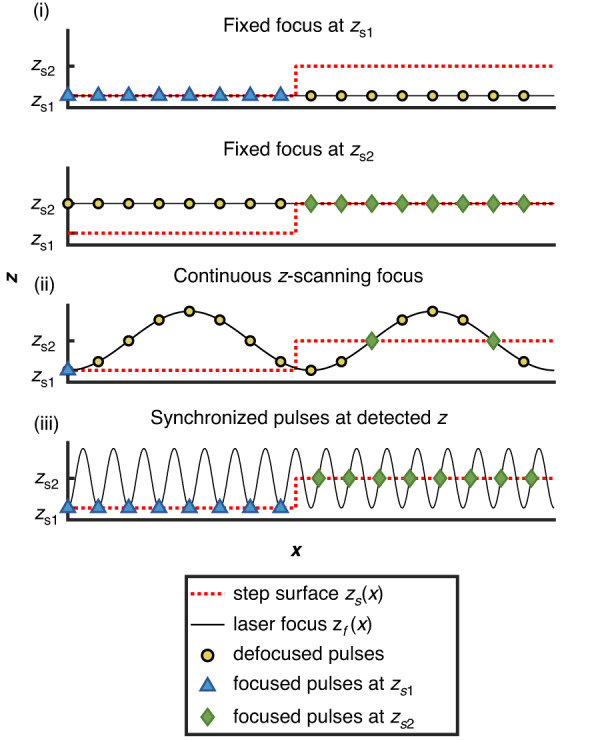
Example of various focusing strategies for laser marking on a step surface with only one lateral translation in $x$ and no motion in $z$. Each subplot shows the same number ($=$16) of pulses. The step surface profile ${z}_{s}(x)$ is in a dashed red line with its low and high surface located at ${z}_{s1}$ and ${z}_{s2}$, and the solid black line plots the laser focal position as a function of $x$. Deposited pulses are represented by markers, with defocused pulses in yellow circles, focused pulses at ${z}_{s1}$ and ${z}_{s2}$ in blue triangles and green diamonds, respectively

We showcase the effectiveness of synchronous focus control for processing non-flat surfaces by comparing various focusing strategies. These strategies involve only one lateral scanning in the $x$ direction, with no motion in the $z$ direction. For example, Fig. 4 plots the surface position ${z}_{s}(x)$ of a step surface (dashed red line, with its low and high surfaces located at ${z}_{s1}$ and ${z}_{s2}$) and the focal position ${z}_{f}(x)$ of the laser pulses (solid black line) for three focusing strategies, namely: (i) fixed focus without the TAG lens, (ii) continuous $z$-scanning focus without synchronization between the laser and the TAG lens, and (iii) synchronized pulses focusing at detected surface $z$ positions. We aim to evaluate the relative amount of focused and defocused pulses out of the same number of total pulses deposited on the step surface. In this context, the term ‘defocused’ refers to cases when the pulse is focused either above or below the target surface, instead of on the target surface as intended. Each subplot in Fig. 4 shows 16 deposited pulses represented by markers, with defocused pulses shown as yellow circles, and focused pulses at ${z}_{s1}$ and ${z}_{s2}$ shown as blue triangles and green diamonds, respectively.

We assume that surface modification can only occur if it is located in the close vicinity of the focus. Using the first strategy, where the laser focus remains fixed and is aligned with either ${z}_{s1}$ or ${z}_{s2}$, only one surface can be modified. Alternatively, if we adopt a continuous $z$-scanning focus, both surfaces can be modified as long as all surfaces are positioned within the scanning range of ${z}_{f}$. However, the absence of surface detection and pulse synchronization results in many continuous scanning pulses not focusing on the surface, thereby becoming ineffective. For example, in Fig. 4 (ii), we plot an oscillating focus pattern with eight pulses per period, and set ${z}_{0}+{z}_{{tag}}={z}_{s1}$ and ${z}_{0}={z}_{s2}$. The periodicity of the pulses is created by sampling the continuous $z$-scanning focus operating at a frequency of ${f}_{{tag}}$ with a laser pulsing at an internal frequency of ${f}_{{laser}}$. The mismatch between the two frequencies would generate an oscillating focus pattern. Only the one pulse at ${z}_{f}(0^{\circ})$ and the two pulses at ${z}_{f}(90^{\circ})$ and ${z}_{f}(270^{\circ})$ are focused per period, while the remaining pulses are defocused and thus do not modify the sample. In this work, we propose the third strategy of using synchronized pulses that are only executed when the scanning ${z}_{f}$ aligns with the detected surface ${z}_{s}$. As shown in Fig. 4 (iii), both surfaces are modified within one lateral translation, and more importantly, all the deposit pulses are effectively focused. Consequently, synchronous focus control proves to be more efficient in delivering entirely focused pulses to non-flat surfaces.

#### Laser marking on a step surface

**Fig. 5:**
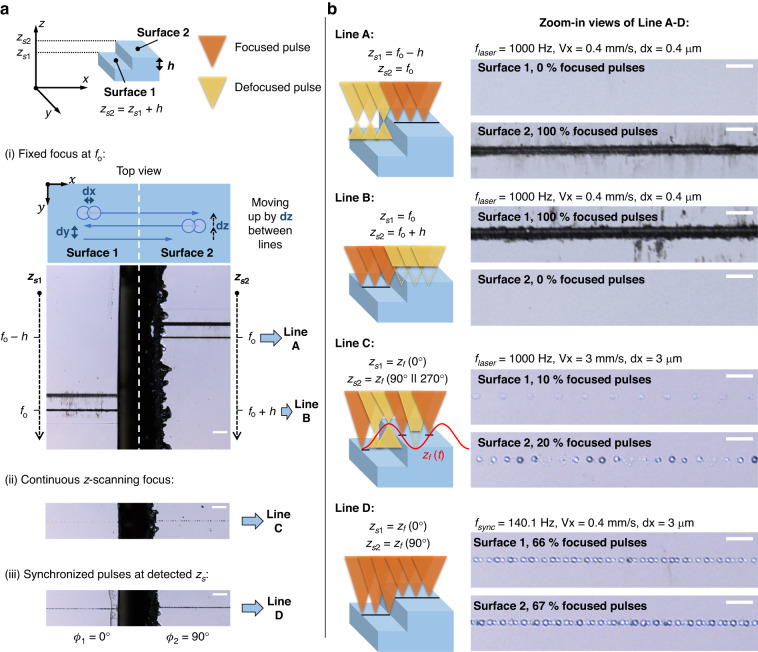
Comparison of fixed focus, continuous *z*-scanning focus, and synchronous focus. **a** Laser marking on a step surface with various focusing strategies. **b** Zoom-in views of the selective marking lines A-D show the percentages of focused pulses over all the deposited pulses on Surface 1 and 2. Focused and defocused pulses are represented by orange and yellow triangles in schematics. The scale bars in **a** and **b** are 100 µm and 30 µm, respectively

We apply the three focusing strategies discussed earlier and compare their processing outputs on a Si step surface. Figure 5a demonstrates the laser marking process on a sample with a step height $h=$ 0.572 mm. We denote the low and high surfaces as Surface 1 and 2, with their $z$ positions at ${z}_{s1}$ and ${z}_{s2}={z}_{s1}+h$, respectively. In all experiments, the laser energy is 5 µJ /pulse. All marking lines are processed with a uniform linear translation speed of ${Vx}$ in the $x$ direction. Similar to the analysis in Fig. 4, we compare the focusing strategies by evaluating the number of focused and defocused pulses on both surfaces, represented by orange and yellow triangles in schematics. Figure 5b provides zoom-in views of the selective marking lines A-D, displaying the percentages of focused pulses relative to all the deposited pulses on Surface 1 and 2. The scale bars in Fig. 5a and b are 100 µm and 30 µm, respectively.

In Fig. 5a (i), we draw 20 marking lines across the step. The laser focus is fixed at ${z}_{f}={f}_{0}$ when in absence of the TAG lens. The fabrication laser is pulsed at ${f}_{{laser}}=$ 1 kHz, and ${Vx}$ is set at 0.4 mm/s. The pulse separation is ${dx}=$ 0.4 µm between adjacent pulses along $x$ and ${dy}=$ 0.1 mm between adjacent lines along $y$. Between two adjacent lines, the sample also moves upward in the positive $z$ direction by ${dz}=$ 0.1 mm using a motorized $z$ stage. Only three of the 20 marking lines modify each surface when the surface is located in the close vicinity of ${f}_{0}$. Line A in Fig. 5b corresponds to the case when all pulses (100%) are focused on Surface 2 but none (0%) on Surface 1, where ${z}_{s2}={f}_{0}$ and ${z}_{s1}={f}_{0}-h$. Similarly, Line B only modifies Surface 1 (100%) but not Surface 2 (0%), where ${z}_{s1}={f}_{0}$ and ${z}_{s2}={f}_{0}+h$. The separated $z$ locations of the marking lines on Surface 1 and 2 suggest that a $z$-axis adjustment of the sample is needed to realign towards the fixed focus when translating from Surface 1 to 2, as the Rayleigh range of the beam ($<$ 200 µm) is smaller than the step height. This experiment represents conventional laser processing without axial detection or focus control, echoing the challenges of re-alignment as discussed in the introduction of this article.

Figure 5a (ii) and (iii) demonstrate two marking lines with continuous $z$-scanning focus (Line C) and synchronized focus control (Line D). Surface 1 is placed at ${z}_{s1}={z}_{0}+{z}_{{tag}}$ for both Line C and D to replicate the scenario in Fig. 4. In synchronous focus control, the laser focal positions are designed to align with the surface $z$ positions, ensuring that ${z}_{f}({\phi }_{1})={z}_{s1}$ and ${z}_{f}({\phi }_{2})={z}_{s2}={z}_{f}({\phi }_{1})+h$. Here, the position of Surface 1, ${z}_{s1}={z}_{0}+{z}_{{tag}}$, corresponds to a triggering phase ${\phi }_{1}=0^\circ$. Using ${z}_{{tag}}=-$0.53 mm and $h=$ 0.572 mm, the calculated triggering phase for Surface 2 is ${\phi }_{2}={\cos }^{-1}(\frac{{z}_{{tag}}\cos \left({\phi }_{1}\right)+h}{{z}_{{tag}}})=94^\circ \approx 90^\circ$. Therefore, for Line D, when the laser is translated from Surface 1 and 2, we switch the triggering phase of the fabrication beam from 0° to 90°. It can be observed that both strategies (ii) and (iii) can mark both Surface 1 and 2 without any mechanical motion in $z$ and within only one lateral translation.

To facilitate a clear comparison between continuous $z$-scanning and synchronous focus control, we have set the pulse separation ${dx}$ to 3 µm for both Line C and D. Note that this particular value of ${dx}$ was chosen solely for the purpose of ensuring an equal number of pulses are physically deposited on each line. The linear translation speed ${Vx}$ is set to 3 mm/s and 0.4 mm/s due to the difference in repetition rate at ${f}_{{laser}}$ and ${f}_{{sync}}$ for Line C and D. Considering the image width of 320 µm along the $x$ direction in Fig. 5b, a total of 320/3 $\approx$ 106 pulses are deposited on each surface. The number of ablated spots is measured by taking central line profiles along $x$. For Line C, only 10% (11 pulses) and 20% (21 pulses) of the total 106 pulses are well focused on Surface 1 and 2. As explained in Fig. 4, the ratio of focused pulses is attributable to the periodicity of the generated focus pattern. Since the $z$-scanning frequency is ${f}_{{tag}}=$ 140.1 kHz and the laser frequency is ${f}_{{laser}}=$ 1 kHz, the frequency ratio ${f}_{{tag}}/{f}_{{laser}}$ creates an oscillating focus pattern with ten pulses per period^42^. Within each ten-pulse period, only the one pulse at ${z}_{f}(0^\circ )$ and the two pulses at ${z}_{f}(90^\circ )$ or ${z}_{f}(270^\circ )$ are focused on Surface 1 and 2. As a comparison, for Line D, 66% (70 pulses) and 67% (72 pulses) of the total deposited pulses are identified on Surface 1 and 2, respectively. The missing pulses ($\approx$ 30%) are solely due to the uncertainty of the triggering signal. Several reference experiments depicted in Fig. s5 of the supplementary information are conducted to count the number of triggered pulses by the TAG lens controller in the absence of the TAG lens in the beam path, reporting a similar missing pulse percentage of 30%. Ideally, all synchronized pulses can be focused onto the measured surface without any mechanical adjustment in $z$ if a more accurate triggering signal is applied.

The comparison of the ratio of focused pulses above suggests that surface detection and focus control by synchronization provide more precise control by reducing the defocused pulses in the continuous $z$-scanning. This leads to two major advantages. First, the processing speed can greatly increase by wisely firing pulses only at the probed surface position. This aspect is particularly relevant for a pulsed laser with a higher repetition rate than the scanning frequency of the TAG lens. Second, the lateral resolution of laser modification is conserved in the synchronized method but may vary in the continuous $z$-scanning method. In Fig. 5, low pulse energy is adopted, so all defocused pulses are below the ablation threshold. In reality, however, the defocused pulse energy can be higher than the ablation threshold, leading to ablation with larger areas and shallower depth than focused pulses. Such an enlarged ablation area is detrimental to lateral resolution^41^. Conversely, the enlarged ablation area in continuous $z$-scanning benefits certain applications thriving for removing more material. For instance, continuous $z$-scanning has been adopted in laser micro-machining for milling more materials^4,42^ and drilling deeper holes^45^ on a flat subtract compared to fixed-focus machining.

### Simultaneous position detection and focus control for auto-focusing

Furthermore, we explore the potential of this technique for auto-focusing and focus control, which requires the simultaneous implementation of in situ position detection and laser refocusing during lateral translation. This auto-focusing process necessitates coordination between multiple hardware and software components. To demonstrate this concept, we first implement real-time detection and subsequently integrate the function of synchronous focus control for auto-focusing.

Supplementary videos (V1 and V2) provide real-time demonstrations of the automatic position detection capabilities of our setup. In the first video (V1), we showcase the detection of periodic back-and-forth movements of the $z$ stage, which is programmed to move at a speed of 0.02 mm/s between $z=$ 0 and $z=$ 0.2 mm. We perform real-time measurements of the moving $z$ stage with two different rates of acceleration and deceleration, 0.002 mm/s^2^ and 0.1 mm/s^2^. Both experiments begin with $z=$ 0.2 mm and go through two cycles of motion. As the programmed $z$ motion is terminated manually, the stage stops at $z=$ 0.2 mm and $z=$ 0.17 mm, respectively.

**Fig. 6:**
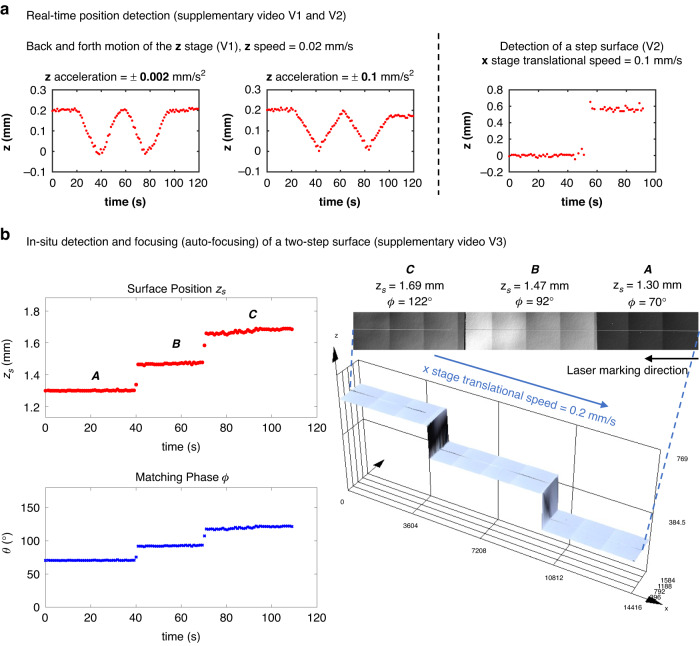
Simultaneous position detection and focus control for auto-focusing. **a** Real-time detected surface position against time at the end of each measurement in supplementary videos V1 and V2. **b** Surface position and matching phase during the auto-focusing laser marking of a two-step surface in supplementary video V3. The three surfaces are labeled by A, B, and C. A 3D microscopy of the two-step surface showing the laser marking line on all three surfaces indicates auto-focusing without any $z$ stage motion. The total marking length shown in the 3D microscope image is 14416 µm

The second video (V2) displays real-time position detection of a step surface as we linearly translate along the $x$ direction, after zeroing the stage at the bottom surface. The $x$ stage translates from 0 mm to $-$7.5 mm with a speed of 0.1 mm/s. The step surface we use here is the same sample studied in Fig. 3 and Fig. 5. Note that in video V2, we use a higher TAG lens frequency (${f}_{{tag}}=$ 350.8 kHz) than in other experiments reported in this paper. Therefore, the oscilloscope displays more than one period of oscillation within its acquisition time of 10 µs. The corresponding calibration condition is labeled as B10 in Table s2. To further illustrate, we include a screenshot of the final measured surface position against time at the end of each measurement in Fig. 6a. The plot shows the detection performance throughout the process.

We further implement real-time in situ position detection and simultaneous focus adjustment as a demonstrator for auto-focusing on a two-step surface (see supplementary video V3). The two-step surface is constructed by adhering three pieces of silicon wafers together. The objective lens used in the dual-laser beam path is a 10$\times$ objective lens. In the video (V3), immediately after turning on the probing beam, we conduct a local calibration experiment by mechanically shifting the $z$ stage in increments of 0.05 mm and acquiring real-time signals from the oscilloscope. A calibration curve similar to those in Fig. 2 is obtained, yielding two fitting parameters: ${z}_{\mathrm{0,1}}=$ 1.51 mm and ${z}_{{tag},1}=-$0.38 mm. The subscript 1 here denotes the axial scanning parameters for the probing beam. Subsequently, we turn on the fabrication beam and characterize its axial scanning parameters following the method described in Section 2.3. The two parameters for the fabrication beam are ${z}_{\mathrm{0,2}}=$ 1.45 mm and ${z}_{{tag},2}=-$0.45 mm, where subscript 2 refers to the fabrication beam parameters. Based on these two sets of calibrated parameters from the probing and fabrication beams, we can calculate the matching phase, $\phi$, in real-time according to the transfer equation in Eq. 5. We then input the matching phase into the TAG lens controller software to trigger the fabrication beam and focus it on the probed surface position. The TAG controller can output three channels of TTL signals at the same time. In the video (V3), we use channel 1 (CH1) and channel 2 (CH2) to trigger the oscilloscope and the fabrication laser, respectively. Due to the limitation in software engineering, the calculated matching phase is manually input to channel 2 of the TAG controller. However, future integration of the detection software with the TAG controller software should automate the input step completely. Figure 6b exhibits the surface position and matching phase during the auto-focusing laser marking of the two-step surface. The three surfaces are denoted as *A*, *B*, and *C*. A 3D microscopy of the two-step surface showing the laser marking line across all three surfaces is also included in Fig. 6b, indicating auto-focusing laser processing without any $z$ stage motion. In the video (V3), we observe process-generated plasma using a laser pulse energy of 5 µJ. Throughout the laser fabrication, we monitor the detected signal in real-time and find that the plasma interference is insignificant and negligible. However, under high laser fluence irradiation, process-generated plasma may cause strong interference in the detected signal. To minimize plasma interference, one can employ time-gated detection by synchronizing the photodetector to measure the signal at specific delays after the fabrication laser pulse and plasma generation. Another option is to utilize polarization or spectral filtering, which allows selective detection of only certain polarized or wavelength components of the signal and effectively suppresses the components corresponding to the plasma emission. Moreover, additional signal processing techniques, such as background subtraction or deconvolution analysis, can be used to separate the desired peaks from the plasma interference.

Our real-time demonstrator achieves a response rate exceeding 1 Hz. However, this is significantly limited by the current hardware and software implementation we used. Ideally, the position data collection and focal control could be completed within a single scanning period of the TAG lens, enabling our method to instantaneously track surface topology without mechanical movement within two periods of $z$-scanning. For instance, when operating the TAG lens at a frequency range of 140–350 kHz, the auto-focusing system could potentially reach a maximum response rate between 70 and 175 kHz.

## Discussion

This article discusses the significance and challenges associated with axial focus alignment in laser processing. Instead of employing separate modules for surface detection and mechanical $z$-axis movements, we propose a novel solution using a single $z$-scanning lens to eliminate slow axial mechanical motions. Our work represents the first single-lens dynamic surface searching approach based on the continuous $z$-scanning of a TAG lens, enabling simultaneous in situ surface detection and focus control.

Our approach adopts an ultrafast varifocal TAG lens in a dual laser beam setup, consisting of a probing beam and a fabrication beam. The probing beam is shaped by the TAG lens and scans continuously along the $z$-axis, and its reflection is detected by a set of confocal optics. We relate the temporal response of the reflected intensity to the probed surface location. Multiple calibrations of the surface detection method are conducted, showing controllable detection ranges and high repeatability. The constructed $z$-scanning detection method has a minimum linearity error of 1.24% with an accuracy of 0.3 µm for a 40$\times$ objective lens, and a minimum linearity error of 1.10% with an accuracy of 15 µm for a 4$\times$ objective lens. We then experimentally demonstrate surface profile measurement of a non-flat step surface. Based on the probed surface position of the step surface, we direct the fabrication beam to the designated positions by synchronizing the fabrication laser at a specific phase of the TAG lens. We adopt synchronized pulses at the probed locations instead of continuous $z$-scanning pulses, which effectively reduces defocused laser pulses, and consequently increases the processing speed and preserves the lateral resolution. In addition, we explore the potential of this technique for auto-focusing with a homemade real-time detection and focusing system. Since the collected position information and the executed focal control are both at 140–350 kHz, our method is designed to instantaneously follow the surface topology without any mechanical movement within two scanning periods of the TAG lens at 70–175 kHz. The results presented pave the way to new methods of material processing for non-flat surfaces or 3D applications through simultaneous auto-focusing and focus control.

## Materials and methods

We have briefly described the methods and the principles of our setup in the Section “Setup and principles”. Figure 1a shows the schematic of the designed optical system, consisting of two beam paths, one for probing marked in green, and the other for fabrication marked in red. The probing laser beam is a low-power continuous wave (cw) green laser at 532 nm (Verdi, Coherent). We design the probing beam path following the principles of confocal optics. The probing laser first passes through a varifocal TAG lens (TLHP, Mitutoyo) and an objective lens (OBJ1). The TAG lens is a liquid lens based on piezoelectricity and liquid resonance, and is powered by a radio frequency (RF) signal from the TAG controller^33,34^. The acoustic standing wave inside the lens creates an oscillating gradient of refractive index so that the TAG lens oscillates between a converging and diverging gradient-index (GRIN) lens of $\approx \pm$ 0.5 diopter at a resonance frequency of 140 kHz^44^. The liquid nature of the lens enables high transmittance in the visible and near-infrared range. Unlike a conventional laser processing setup with a fixed focus at ${z}_{0}$, the focal position of the probing beam ${z}_{f}$ continuously oscillates as a function of time $t$ with the inclusion of the TAG lens, as plotted in the inset of Fig. 1a. The probe beam is reflected from the surface of the workpiece and is directed back to the objective lens (OBJ1). A second objective lens (OBJ2 $=$ 10$\times$, NA $=$ 0.25) is used to refocus the reflected beam after passing through the beam splitter. A homemade pinhole with a diameter of 30 µm is placed at the focus of OBJ2 to filter out-of-focus light. The pinhole is prepared by femtosecond laser ablation of a copper foil. The reflected beam passing through the pinhole is detected by a biased photodiode (PD, Thorlabs). The photodiode signal is first measured by an oscilloscope (Scope, Tektronix), and then transferred in real-time to the computer for processing. Based on the temporal response of the detected PD signal, we can calculate the surface position ${z}_{s}$ for the probed site according to Eq. 4. The fabrication laser is a femtosecond laser at 800 nm (Solstice Ace, Spectra-Physics, 1 kHz). The fabrication beam path is similar to the setup for multiple-focal laser processing of transparent materials in our previous work^44^.

The real-time demonstrators in Video V1, V2 and V3 consist of an oscilloscope, a programmable mechanical stage and a computer running Matlab and the TAG lens controller software. After the probing beam is turned on, the photodiode signal is first acquired by the oscilloscope. The length of acquired waveforms is 1000 points, with an acquisition time of 10 µs in V1 and V2, and 20 µs in V3. The oscilloscope is triggered on the falling edge of a TTL signal sent from channel 1 of the TAG controller. The acquired waveform is then transferred to Matlab using the Instrument Control Toolbox via the Virtual Instrument Standard Architecture (VISA) interface. The signal processing includes (1) noise filtering by the *lowpass* function with a passband frequency of $2{f}_{{tag}}$, and (2) peak detection by the *findpeaks* function. We can then obtain the time interval from the identified double peaks, and subsequently calculate the surface position and the matching phase based on the calibrated ${z}_{0}$ and ${z}_{{tag}}$.

In our experiments, non-flat surfaces are created by sticking silicon wafers together. The step surface in Figs. 3, 5, and 6a is constructed by adhering two silicon wafers of 500 µm thickness with a thin layer of optical glue. The two-step surface in Fig. 6b is constructed by adhering two pieces of silicon wafers of 200 µm thickness to a silicon wafer of 500 µm thickness with a thin layer of optical glue. Optical images in Figs. 3, 5 and 6 are taken with a laser confocal microscope (Olympus LEXT OLS4000).

### Supplementary information

Supplementary information

Supporting Video V1

supporting Video V2

Supporting Video V3

## Data Availability

The authors declare that the data supporting the findings of this study are available within the paper and its supplementary information files. Additional data are available from the corresponding author upon request.
