# Supplementary material for: Single-lens dynamic z-scanning for simultaneous in situ position detection and laser processing focus control
Source: Light Sci Appl. 2023 Nov 17;12:274. doi: 10.1038/s41377-023-01303-2 (PMC10656504; doi:10.1038/s41377-023-01303-2)
Supplement: Supplementary file 1 — Supplementary information [file 41377_2023_1303_MOESM1_ESM.pdf]

Supplementary Information for Single-lens dynamic z-scanning for simultaneous in-situ position detection and laser processing focus control

Xiaohan Du[1,2], Camilo Florian[3,4], and Craig B.Arnold[1,4,*]

[1]Department of Mechanical and Aerospace Engineering, Princeton University, Princeton, NJ, 08544, USA
[2]Department of Systems Engineering, City University of Hong Kong, Hong Kong, China
[3]Institut für Werkstofftechnik, Universität Kassel, 34125, Kassel, Germany
[4]Princeton Materials Institute, Princeton University, Princeton, NJ, 08544, USA
[*]Corresponding Author, email: *cbarnold@princeton.edu.*

1 Ray transfer matrix analysis

We can use a simplified ray transfer matrix model to calculate the photodiode signal. The outgoing ray, r_p, which is focused at the pinhole can be expressed by,

$$r_p = L_p \cdot F_2 \cdot L_2 \cdot F_1 \cdot L_1 \cdot r_{in} \tag{s1}$$

where

$$L_i = \begin{bmatrix} 1 & l_i \\ 0 & 1 \end{bmatrix}, F_i = \begin{bmatrix} 1 & 0 \\ -\frac{1}{f_i} & 1 \end{bmatrix}. \tag{s2}$$

l_i is the distance between optical elements, f_i is the focal length of the objective lens, and r_{in} denotes the incoming ray that is reflected from the sample surface. Due to the dynamic scanning of the probing beam, l_1 is a function of time t.

$$l_1(t) = z_0 + z_{tag}\cos(2\pi f_{tag} t) + \Delta z \tag{s3}$$

where Δz is the displacement of the sample surface from the mean value of z-scanning (z_0). The distance of the focused beam after the second objective lens is denoted by l_p, and is solved from Equation s3 as,

$$l_p(t) = \frac{l_2 + (1 - \frac{l_2}{f_1})l_1(t)}{\frac{l_2}{f_2} - 1 + (\frac{1}{f_2} + \frac{1 - \frac{l_2}{f_2}}{f_1})l_1(t)} \tag{s4}$$

The radius of the beam at the pinhole R_p is then [1],

$$R_p^2 = R_0^2[(\frac{\lambda}{\pi R_0})^2(l_3 - l_p(t))^2 + 1] \tag{s5}$$

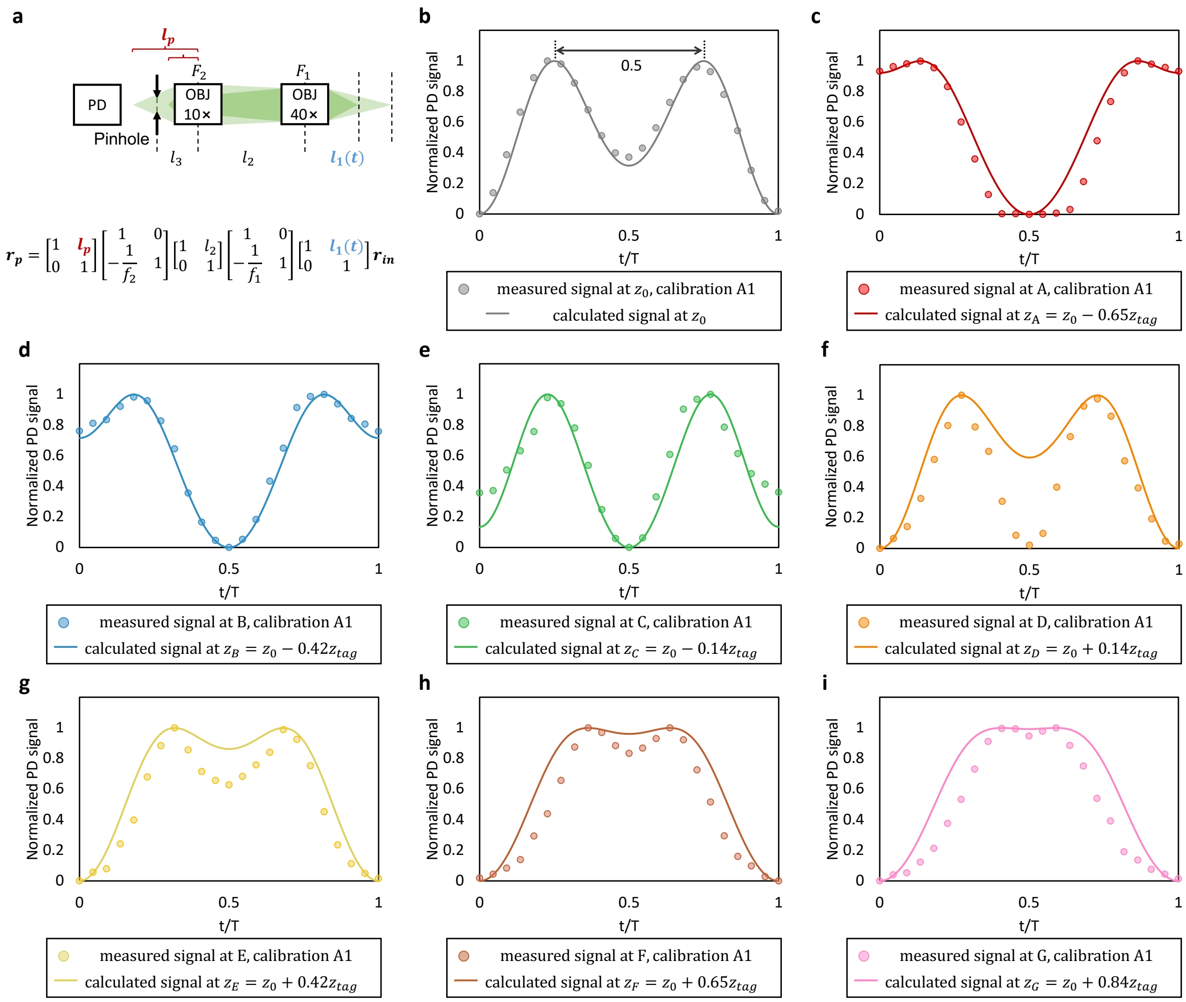

Figure s1: a. A simplified ray transfer matrix model for the probing beam. b. Comparison between the measured signal at $z_s = z_0$ from calibration A1 and the calculated signal by the ray transfer matrix model. c-i. Calculated and measured photodiode signal for locations A to G in Figure 1b-c of the main text.

where R_0 is the minimum beam radius for the beam focused at $l_p(t)$, l_3 is the distance between the pinhole and the second objective lens. We assume that R_0 is constant despite the varying optical power of the TAG lens. Lastly, the intensity of the photodiode signal passing through the pinhole is considered proportional to $1/R_p^2$ [1]. Therefore the photodiode signal can be obtained by substituting Equation s4 to Equation s5.

Figure s1b plots the calculated photodiode signal at $z_s = z_0$ (or $\Delta z = 0$) in a dashed line. We have plugged in the following values to all the constants: $f_1 = z_0 = 4$ mm, $f_2 = 16$ mm, $l_2 = 160$ mm, and $z_{tag} = 11.98$ μm from calibration experiment A1. The time interval between double peaks in the calculated signal is exactly $0.5T$. The dots show the measured signal from calibration experiment A1, which is in good agreement with the calculated signal. Similarly, Figure s1c-i compares the calculated and the measured signal for locations A to G (see Figure 1b-c of the main text) within the scanning range from calibration experiment A1.

2 List of calibration measurements

We evaluate the accuracy and the repeatability of the in-situ detection method under different alignment and driving conditions. Multiple calibration experiments (A1-A9 and B1-B10) are conducted with OBJ1 = 40× and OBJ1 = 4×, respectively. Table s1 and Table s2 exhibit the setting parameters (power of probing beam P_{pb}, input voltage V_{tag}, and scanning frequency f_{tag}), the fitted value of z_0 and z_{tag}, as well as the calculated accuracy and linearity error of the calibration experiments. Figure s2 and Figure s3 exhibit the data points for the calibration experiments. We plot the measured surface position z'_s versus the real surface position z_s in red, with the fitted intercept (z_0) and slope (z_{tag}). The secondary axis plots the error $\delta z = z'_s - z_s$ in blue.

Table s1: List of all calibration measurements with OBJ1 = 40×

No.	P_{pb} (W)	V_{tag} (V)	f_{tag} (kHz)	z_0 (mm)	z_{tag} (μm)	Accuracy max $\lvert\delta z\rvert$ (μm)	Linearity error ε (%)
A1	0.06	15	139.8	2.4482	11.98	0.31	1.30
A2	0.06	15	139.8	2.4484	11.56	0.41	1.77
A3	0.06	15	140.5	2.4483	11.54	0.30	1.29
A4	0.06	15	140.5	2.4484	12.18	0.48	1.95
A5	0.25	15	140.0	2.4481	11.10	0.30	1.35
A6	0.25	15	140.3	2.4482	12.13	0.30	1.24
Average of A1-A6				2.4483	11.75	0.35	1.49
SD of A1-A6				0.0001	0.420	0.08	0.30
A7	0.25	9	140.4	2.4482	7.400	0.23	1.57
A8	0.25	9	140.4	2.4482	7.408	0.19	1.25
A9	0.06	18	139.2	2.4481	13.47	0.42	1.57

Table s2: List of calibration measurements with OBJ1 = 4×

No.	P_{pb} (W)	V_{tag} (V)	f_{tag} (kHz)	z_0 (mm)	z_{tag} (mm)	Accuracy max δz (mm)	Linearity error ε (%)
B1	0.06	15	141.0	2.187	0.719	0.034	2.37
B2	0.06	15	141.0	2.173	0.730	0.022	1.52
B3	0.06	15	141.0	2.150	0.736	0.039	2.68
B4	0.06	15	141.1	2.167	0.654	0.017	1.28
B5	0.25	15	141.1	2.169	0.664	0.030	2.29
B6	0.25	15	141.1	2.174	0.665	0.015	1.10
Average of B1-B6				2.170	0.695	0.026	1.87
SD of B1-B6				0.012	0.037	0.010	0.66
B7	0.06	18	141.0	2.101	0.819	0.044	2.71
B8†	0.06	15	140.4	2.202	1.365	0.169	6.19
B9†	0.06	15	140.4	2.182	1.374	0.158	5.73
B10	0.11	15	350.8	2.219	1.058	0.052	2.48

† pinhole removed

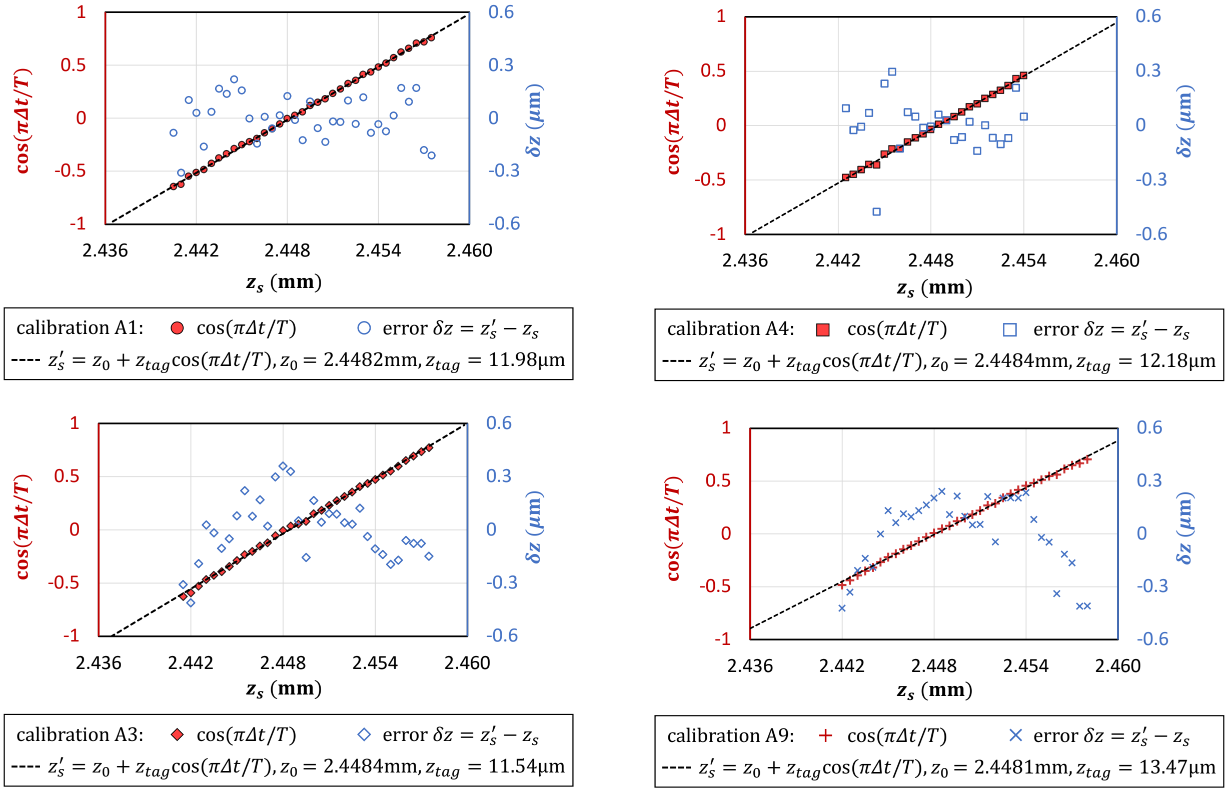

Figure s2: Data for calibrations with OBJ1 = 40×, showing a plot of the measured surface position z'_s versus the real surface position z_s in red, with the fitted intercept (z_0) and slope (z_{tag}). The secondary axis plots the error $\delta z = z'_s - z_s$ in blue.

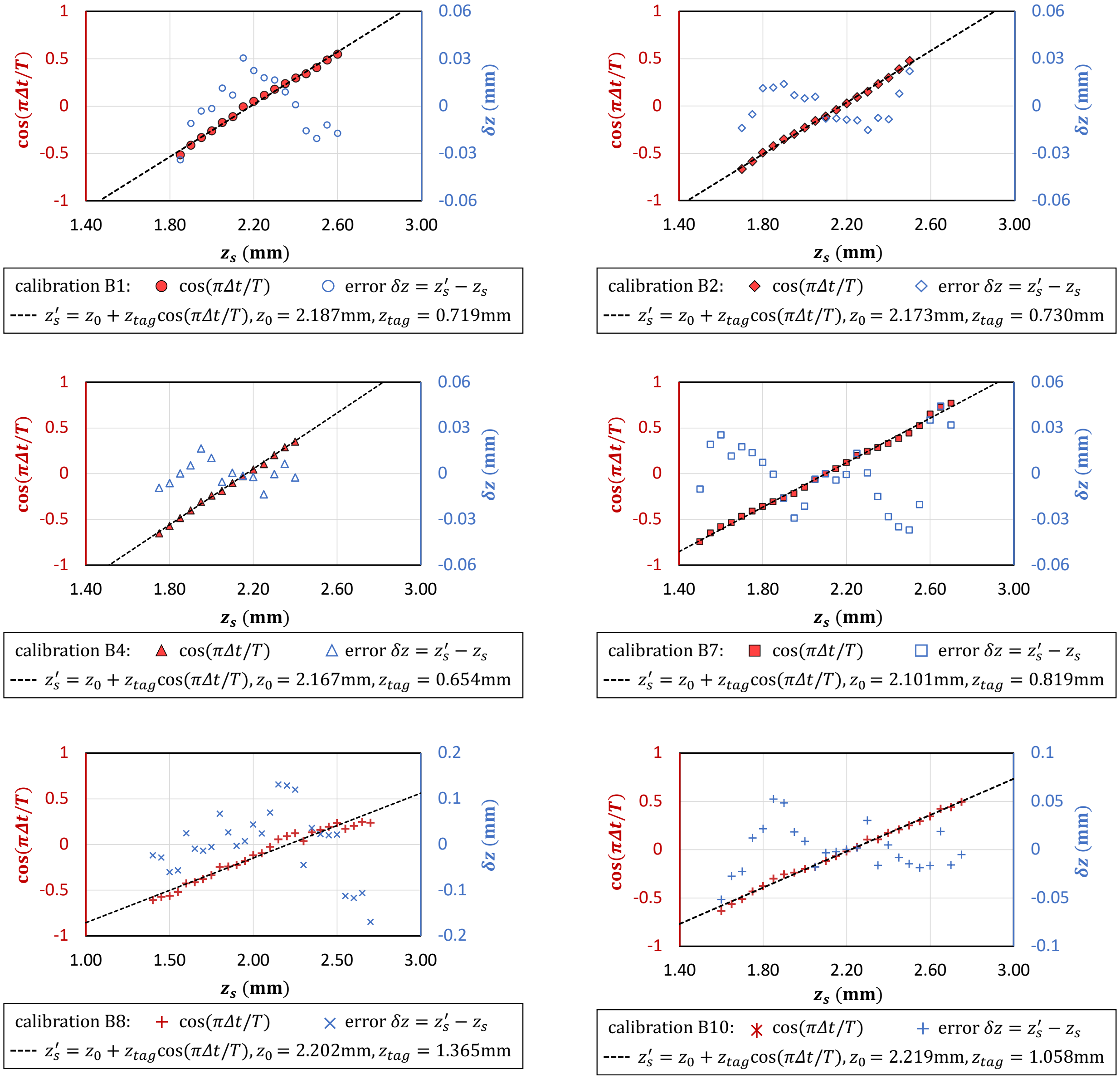

Figure s3: Data for calibrations with OBJ1 = 4×, showing a plot of the measured surface position z'_s versus the real surface position z_s in red, with the fitted intercept (z_0) and slope (z_{tag}). The secondary axis plots the error $\delta z = z'_s - z_s$ in blue.

3 Characterization of synchronous pulses

Figure s4a shows the characterized focal position $z_f(\phi)$ triggered at various phases ϕ from 0° to 180°. $z_f(\phi)$ is measured as the z location of the thinnest line from a set of z-scan line ablation experiment on a Si wafer using a 10× objective lens, $f_{tag} = 140.1$ kHz, and $V_{tag} = 15$ V [2]. The adopted laser energy is 5 μJ (± 25%) per pulse with a linear translation speed of 0.65 mm/s. The positive z direction is opposite to the beam propagation direction. The error bar is set as ± 0.1 mm, which is the z stage motion pitch. The solid line represents a least-square fit of $z_f(\phi)$ based

on Equation 7 in the main text with $R^2 = 0.96$. The fitted $z_{tag} = -0.53$ mm and $z_0 = f_0 - 0.27$ mm, where f_0 is the focal length of the objective lens. The dashed line plots an envelope of $z_f(\phi)$ accounting for any uncertainty originating from the triggering pulse width by adding a $\pm\pi f_{tag}\delta t$ term to its phase. The envelope of focal position is expressed as $z_f(\phi) = z_0 + z_{tag}cos(\phi \pm \pi f_{tag}\delta t)$. Thus, the accuracy of synchronous focus control is defined as the axial width of the envelope at 90°. For $z_{tag} = -0.53$ mm, the maximum axial width at $\phi = 90^o$ is $\pm$ 0.22 mm if $\delta t = 1$ μs, and $\pm$ 0.023 mm if $\delta t = 100$ ns.

Figure s4b shows the measured ablated line depth using pulses triggered at $\phi = 0^o$, 90° and 180°. The peaks indicate the focal positions at $f_0 - 0.8$, $f_0 - 0.1$, and $f_0 + 0.2$ mm respectively. The corresponding ablation depth when at focus decreases from 0.95 μm to 0.22 μm as the lens power decreases from $\phi = 180^o$ (the most converging) to $\phi = 0^o$ (the most diverging).

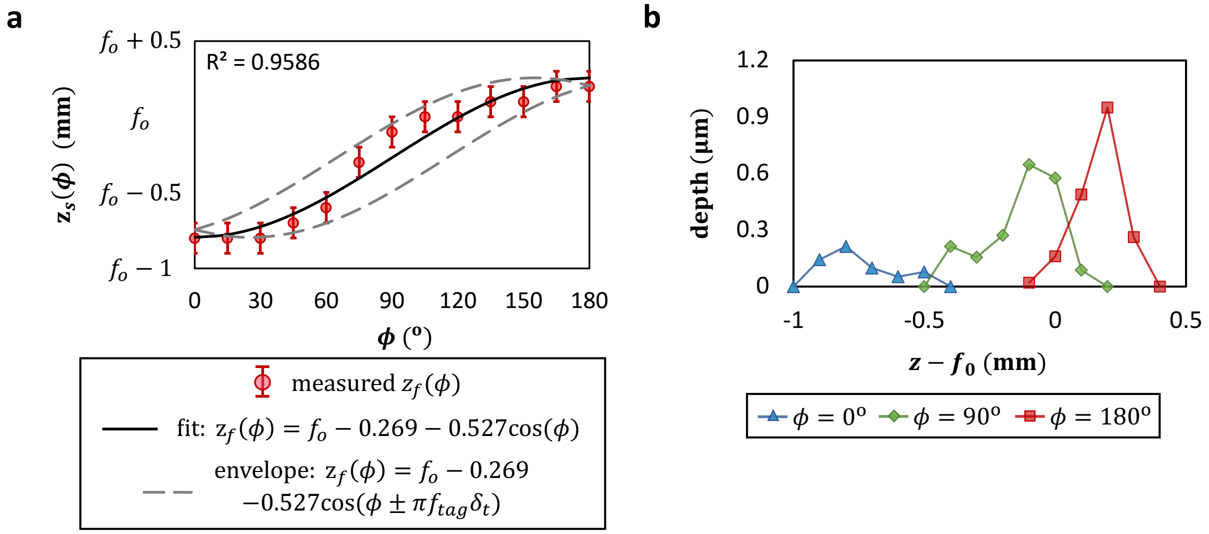

Figure s4: a. Characterized focal position $z_f(\phi)$ as a function of the triggering phase ϕ by z-scan experiment on Si wafers. The solid line plots a least-square fit, and the dashed line draws an envelope including any uncertainty originating from the triggering pulse width. b. Measured ablated depth on Si with laser pulses triggered at $\phi = 0^o$, 90° and 180°.

4 The reference experiment on synchronized pulses

Several reference experiments are performed to determine the number of triggered pulses by the TAG lens controller in the absence of the TAG lens in the beam path. The laser is triggered at a single phase in Figure s5, with linear translation speeds Vx = 1.2 mm/s and Vx = 2.4 mm/s, presented in two columns. The TAG lens frequencies for the four rows were 140.1 kHz, 140.9 kHz, 141.1 kHz, and 283.4 kHz, corresponding to f_{sync} values of 140.1 Hz, 140.9 Hz, 141.1 Hz, and 283.4 Hz, respectively. With an image length of 0.64 mm, the calculated number of triggered pulses displayed in the image is $0.64/(Vx/f_{sync})$. To illustrate the results, we marked the ratio between the counted number of pulses and the calculated number of pulses on the images, revealing a missing percentage of around 30%.

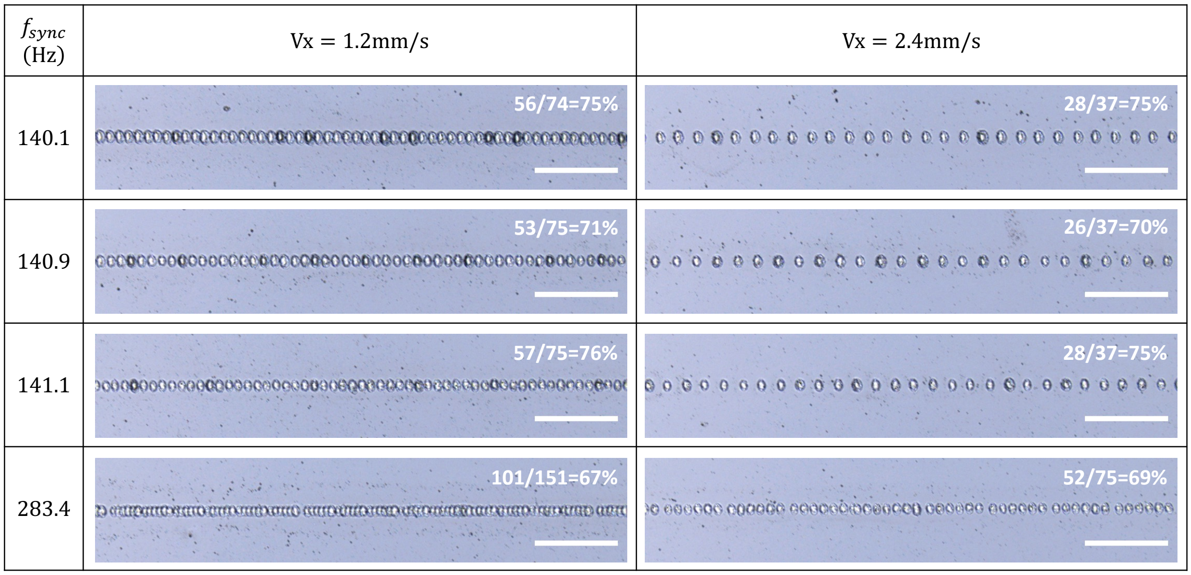

Figure s5: Reference experiments of synchronous pulses. The image length is 0.64 mm, and the scale bar is 100 μm.

5 Supplementary videos for the real-time detection and focusing

Figure s6a illustrates the video frames that depict the setup of the real-time detection system in supplementary videos V1 and V2. The system comprises an oscilloscope, a programmable xyz stage, and a computer running Matlab. Frame 1-4 corresponds to the sequential steps taken to initiate data acquisition. In the first video (V1), we detect programmed periodic back-and-forth motions of the z stage, which translates between 0 mm and 0.2 mm with varying acceleration/deceleration. Figure s6b displays the selected frames 5-8 after the frames 1-4 in Figure s6a for z motion with a velocity of 0.02 mm/s and an acceleration of 0.002 mm/s^2. On the other hand, Figure s6c illustrates the selected frames for z motion with a velocity of 0.01 mm/s and an acceleration of 0.1 mm/s^2. Both experiments begin with $z = 0.2$ mm and go through two cycles of motion. As the

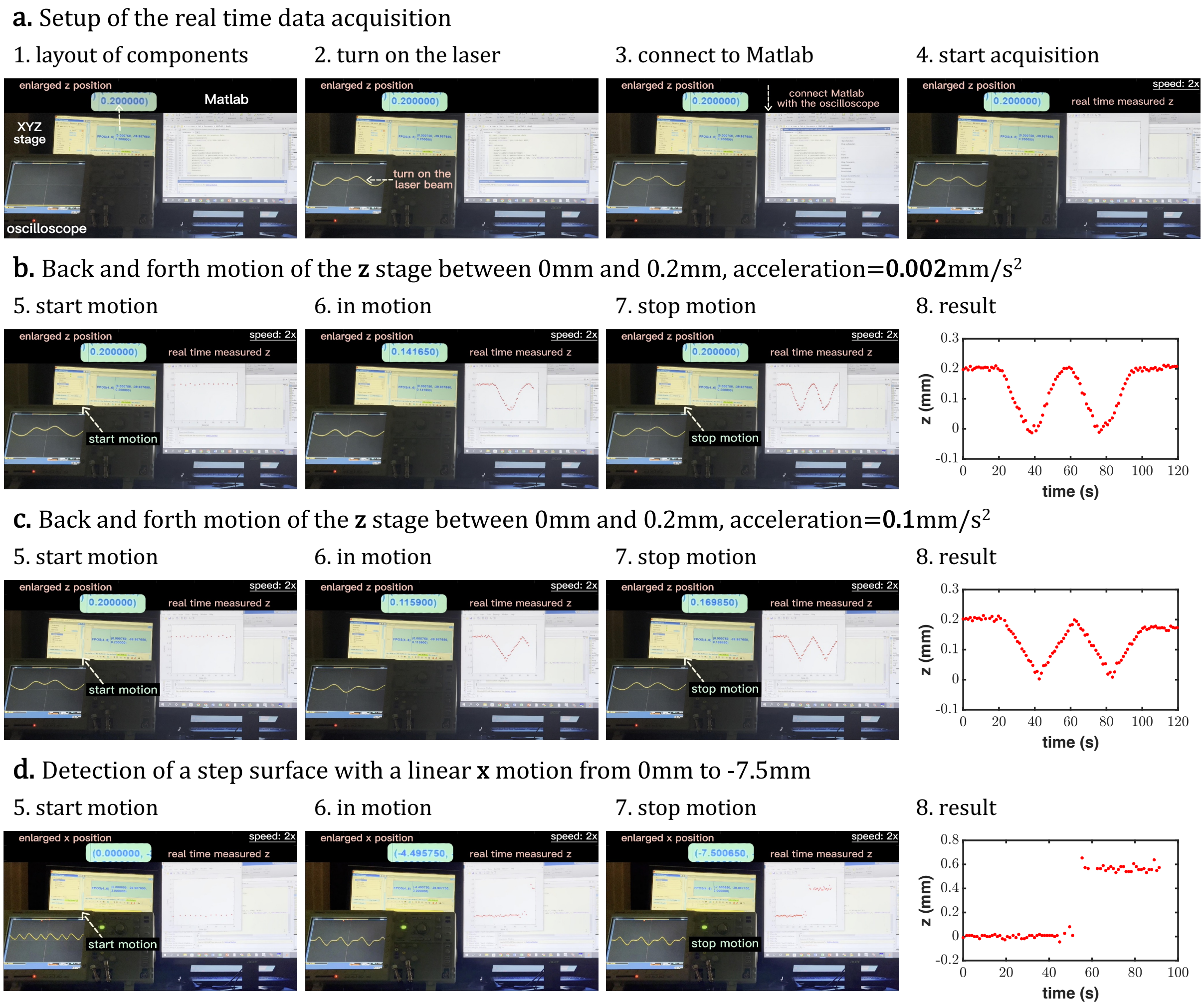

Figure s6: Video frames showing real-time detection. a. Frame 1-4, showing the setup and the sequential steps taken to start data acquisition for both Video V1 and V2. b-c. Frame 5-8 of Video V1, showing the back-and-forth motion of the z stage with different accelerations. d. Frame 5-8 of Video V2, showing the detection of a step surface with a linear motion in x.

programmed z motion is terminated manually, the stage stops at $z = 0.2$ mm and $z = 0.17$ mm in Figure s6b and c, respectively, as shown in Frame 7. Frame 8 plots the final measured z_s versus time at the completion of each measurement. In the second video (V2), we detect the step surface in real-time while translating linearly along the x direction from 0 mm to -7.5 mm. The x stage speed is 0.1 mm/s. Figure s6d displays the selected frames 5-8 for a x motion with a velocity of 0.1 mm/s. The measured step size is 0.565 mm, with a standard deviation of 0.024 mm (determined by the top surface variation).

In the third video (V3), we implement real-time position detection and simultaneous focusing for laser marking of a two-step surface. Selected frames of video V3 are exhibited in Figure s7. The auto-focusing setup consists of multiple hardware and software components, as displayed in Frame 1 and 2. After turning on the probing beam in Frame 3), we conduct a local calibration experiment by mechanically shifting the z stage for 0.05 mm per step and acquiring the real-time

signal from the oscilloscope, as shown in Frame 4. Frame 5 plots the obtained calibration curve, with the two fitting parameters as $z_0 = 1.51$ mm and $z_{tag} = -0.38$ mm. Next, based on the two sets of calibrated parameters of the probing and fabrication beams, the matching phase ϕ can be calculated in real-time accordingly. We then input the matching phase into the TAG lens controller software to trigger the fabrication beam and focus on the probed surface position (Frame 6). Frame 7-9 shows that by adjusting the matching phase to trigger the fabrication laser, we can focus on different surfaces while the x stage moves with a velocity of 0.2 mm/s.

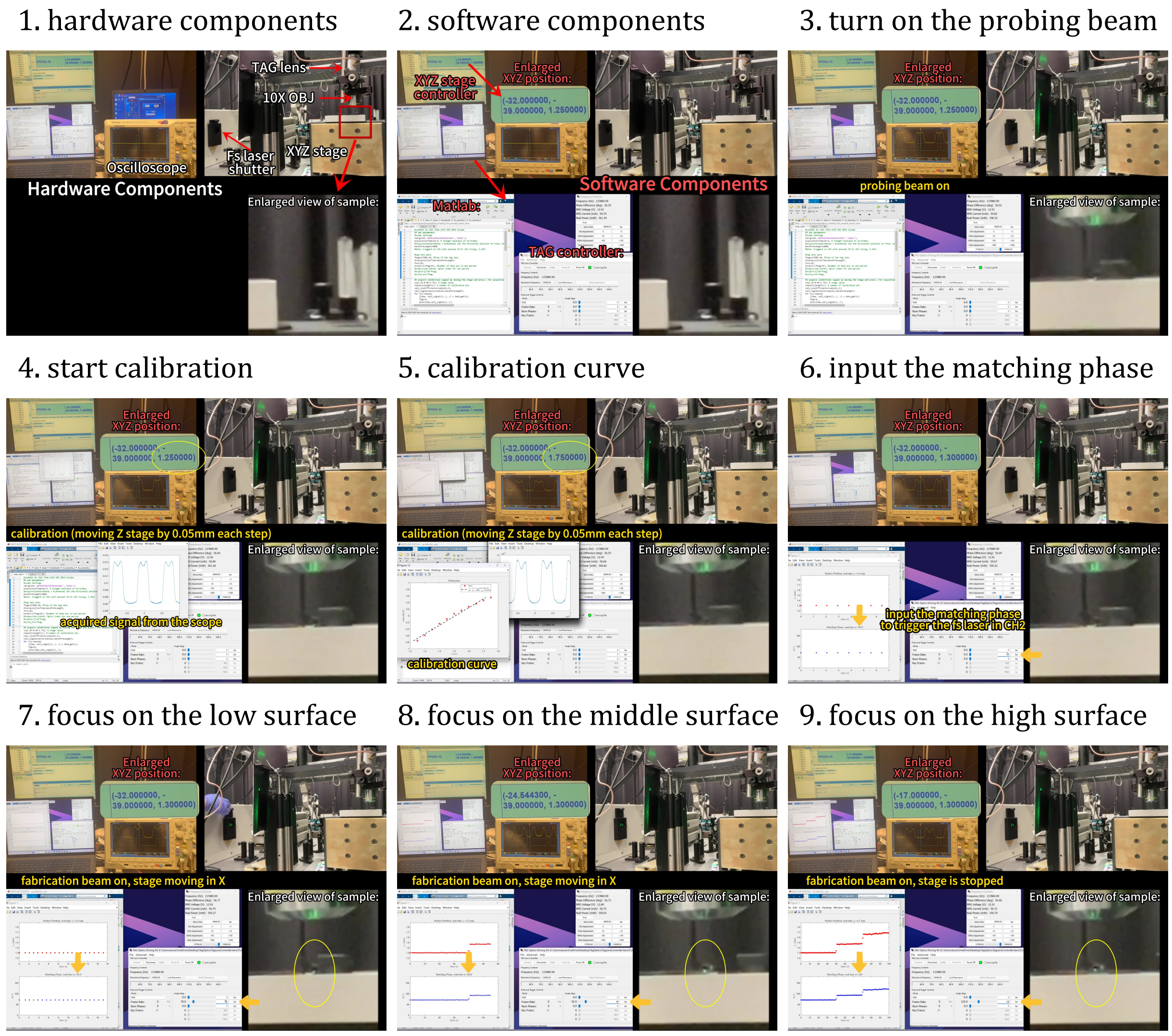

Figure s7: Video frames showing real-time detection and simultaneous focusing of a two-step surface.

Supplementary information accompanies the manuscript on the Light: Science & Applications website (http://www.nature.com/lsa).

References

[1] Nakazawa, K. *et al.* Confocal laser displacement sensor using a micro-machined varifocal mirror. *Applied Optics* **56**, 6911 (2017).

[2] Du, X., Florian, C. & Arnold, C. B. Parametric study of multi-focal laser processing using an ultrafast tunable acoustic lens. In *Laser Applications in Microelectronic and Optoelectronic Manufacturing (LAMOM) XXVII*, vol. 11988, 94–98 (SPIE, 2022).
